# Intra-arterial delivery of neurospheres into isolated perfused porcine colons: a proof of concept

**DOI:** 10.1093/biomethods/bpae022

**Published:** 2024-04-02

**Authors:** Richard D Martel, Nicolas A Hoyos, María Ángeles Tapia-Laliena, Irmgard Herrmann, Martin Herrmann, Rasul Khasanov, Karl-Herbert Schäfer

**Affiliations:** Department of Pediatric Surgery, Medical Faculty Mannheim, University Medical Center Mannheim, Heidelberg University, Heidelberg, Germany; Department of Neurophysiology, Mannheim Center for Translational Neurosciences (MCTN), Medical Faculty Mannheim, Heidelberg University, Heidelberg, Germany; Department of Pediatric Surgery, Medical Faculty Mannheim, University Medical Center Mannheim, Heidelberg University, Heidelberg, Germany; Department of Pediatric Surgery, Medical Faculty Mannheim, University Medical Center Mannheim, Heidelberg University, Heidelberg, Germany; Department of Medicine 3, Universitäts-Klinikum Erlangen, Erlangen, Germany; Department of Pediatric Surgery, Medical Faculty Mannheim, University Medical Center Mannheim, Heidelberg University, Heidelberg, Germany; Department of Medicine 3, Universitäts-Klinikum Erlangen, Erlangen, Germany; Deutsches Zentrum Immuntherapie DZI, Universitätsklinikum Erlangen, Erlangen, Germany; Department of Pediatric Surgery, Medical Faculty Mannheim, University Medical Center Mannheim, Heidelberg University, Heidelberg, Germany; Enteric Nervous System Group, University of Applied Sciences Kaiserslautern, Zweibrücken, Germany

**Keywords:** cell therapy, intra-arterial, neurosphere, Hirschsprung disease, enteric nervous system

## Abstract

Cell replacement in aganglionic intestines is a promising, yet merely experimental tool for the therapy of congenital dysganglionosis of the enteric nervous system like Hirschsprung disease. While the injection of single cells or neurospheres to a defined and very restricted location is trivial, the translation to the clinical application, where large aganglionic or hypoganglionic areas need to be colonized (hundreds of square centimetres), afford a homogeneous distribution of multiple neurospheres all over the affected tissue areas. Reaching the entire aganglionic area *in vivo* is critical for the restoration of peristaltic function. The latter mainly depends on an intact nervous system that extends throughout the organ. Intra-arterial injection is a common method in cell therapy and may be the key to delivering cells or neurospheres into the capillary bed of the colon with area-wide distribution. We describe an experimental method for monitoring the distribution of a defined number of neurospheres into porcine recta *ex vivo,* immediately after intra-arterial injection. We designed this method to localize grafting sites of single neurospheres in precise biopsies which can further be examined in explant cultures. The isolated perfused porcine rectum allowed us to continuously monitor the perfusion pressure. A blockage of too many capillaries would lead to an ischaemic situation and an increase of perfusion pressure. Since we could demonstrate that the area-wide delivery of neurospheres did not alter the overall vascular resistance, we showed that the delivery does not significantly impair the local circulation.

## Introduction

Hirschsprung disease is the most frequent representative of congenital enteric neuropathies. It occurs in 1:5000 newborns and is due to the absence of ganglia cells in a part of the enteric nervous system. It is characterized by the absence of ganglia over a highly variable length of the colon, however, always including the most aboral segment of the rectum. Hirschsprung disease is currently treated by surgical intervention during the newborn period. However, alternative therapeutic options are much needed due to high perioperative morbidity and often unsatisfactory functional results [1, 2].

For regeneration of the enteric nervous system, many studies have examined transplantation success and functional integration of grafts like pluripotent stem cells or neurospheres by direct injection into the intestinal wall. These studies serve as a proof of concept for enteric nervous system regeneration [3]. However, the currently applied techniques do not have the potential to reach the entire length of the aganglionic section of the colon with homogenously positioned grafts [4–9]. Especially in the aganglionic colon, the migration of the graft is reduced [10]. Another factor that is disregarded when rodents are used as test organisms is the anatomical dimension in humans; aside from large differences in gut physiology between species. Therefore, we aimed at the proof of concept of delivering enteric neurospheres by vascular injection in an *ex vivo* model with dimensions similar to those of humans. An organ bath arrangement with microprocessor-controlled perfusion and aeration by an oxygenator allows one to observe the vitality of the porcine colon segments (rectum) which is indicated by their motor activity. Our technical approach aimed at achieving a homogeneous dispersion of neurospheres in the colon and to localize grafting sites of single neurospheres suitable for precise biopsies without impairing organ perfusion. Using this approach for *in-vivo* transplantation studies will not only pave the road for clinical application but also reduce animal experiments by establishing this *ex vivo* model.

## Materials and methods

### Animals and tissue preparation

This study was carried out in strict accordance with the recommendations for the care and use of laboratory animals of the German animal protection law. Animal experiments were approved by the Regierungspräsidium Baden-Württemberg, Germany and the Veterinary Inspection Office in Mannheim, Germany. Authorization number for experiments on pigs: 35-9185.81/G-78/18. Authorization number for experiments on rats: I-29/23 R.

Pigs underwent experiments lasting for 6.5–8.5 h under general anaesthesia by a collaborating group. The pigs were pre-medicated with i.m. injection of azaperone (Stresnil, Janssen Pharmaceutica, Beerse, Belgium) 4 mg/kg, and midazolam 1 mg/kg. General anaesthesia was induced with Propofol (Fresenius, Bad Homburg, Germany) 1–2 mg/kg i.v., and maintained with pentobarbital (Narcoren, Merial, Halbergmoos, Germany) 12 mg/kg bolus followed by 8–14 mg/kg/h. We intubated, ventilated, and monitored the vital parameters: ECG, EtCO_2_, SPO_2_, and rectal temperature.

**Figure 1.**
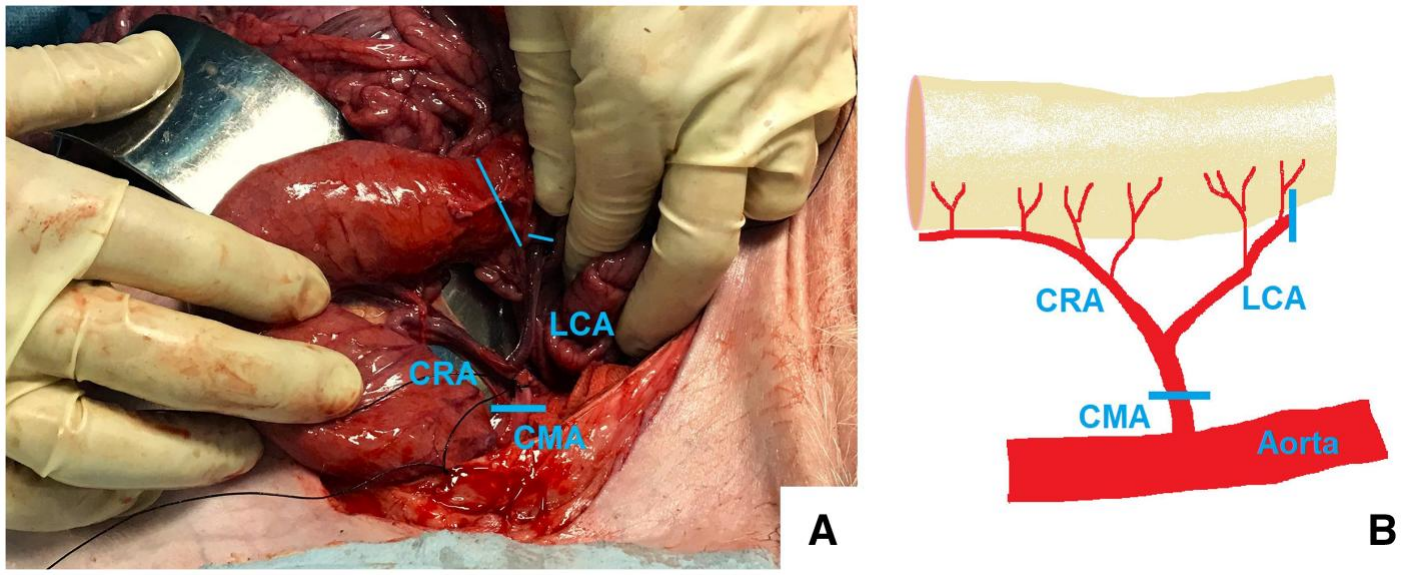
Intraoperative situs during preparation of the porcine rectum and schematic of the vascular anatomy and resection levels. (A) In pigs, a formal inferior mesenteric artery is not expressed. There is a relatively small mesenteric artery supplying the rectum and a short part of the distal colon called caudal mesenteric artery or posterior mesenteric artery. For our preparation, the rectum was released from the retroperitoneal space and the cranial rectal artery (CRA) and vein prepared. The oral resection level at the rectum (long oblique line) corresponded to the vascularization following the resection level of the left colic artery (LCA). The distal resection level was just as aboral as possible on the level of the pelvic floor. The preparation included a short stump of the caudal mesenteric artery (CMA). The resection level at the CMA is shown by the inserted horizontal line. (B) The figure illustrates the intraoperative situs: 1. Cranial rectal artery (CRA), left colic artery (LCA), and caudal mesenteric artery (CMA).

An approximately 6-cm skin incision was made medially at the hindlimb and the saphenous nerve was exposed. Single action potentials could be recorded time-locked upon square wave pulses (width 0.5 ms) administered with 20 mA through a pair of non-insulated tungsten electrodes (FHC, Bowdoinham, Main) [11]. The experiments were terminated by euthanasia with cardioplegia by potassium chloride. Immediately after confirmation of death by a veterinarian, the abdomens were opened. A 15-cm long rectum segment was dissected together with its mesorectum containing the cranial rectal artery, a 2-cm segment of the left colonic artery and a short segment of the caudal mesenteric artery from which the latter vessels originate (Fig. 1A and B) [12]. The specimens were kept in Eagle’s Minimal Essential Medium (MEM) with 100 U/ml Penicillin and 100 µg/ml Streptomycin (Life Technologies Europe BV, Bleiswijk, the Netherlands) on ice for transport and preparation.

The preparation process included: (i) cannulation of the caudal mesenteric artery and vein with each a 20G infusion cannula, (ii) ligation of the vessels at both ends of the specimen, (iii) wash-out of the vasculature with MEM-Eagle (Biological Industries, Kibbutz Beit-Haemek, Israel) enriched with 200 U/ml Heparin (Ratiopharm, Ulm, Germany). To generate neurospheres, we employed small intestine samples from week-old Sprague Dawley rats (Janvier Labs, Le Genest-Saint-Isle, France). They were harvested post-mortem after euthanasia by decapitation.

### Organ perfusion

The organ bath arrangement comprised an outer incubator and an acryl glass container filled with Krebs Henseleit solution (Sigma-Aldrich Chemie GmbH, Taufkirchen, Germany). The bath was gassed with Carbogen (Linde, Germany). Vascular perfusion with MEM was performed employing a Purema polyethersulfone fibre oxygenator (Medica, Medolla, Italy) gassed with Carbogen and equipped with a heat exchanger set to 37°C. The arterial pressure was maintained at a level of 40 mmHg by an Ismatec Ecoline VC-MS peristaltic pump (Cole-Parmer GmbH, Wertheim, Germany) which was servo-controlled by a Plugsys modular system (Hugo Sachs Elektronik—Harvard Apparatus GmbH, March, Germany). Venous outflow pressure was adjusted to 4 cmH_2_O by a riser tube 4 cm long above the bath level. The vascular medium was recirculated using a 500 ml reservoir. The resolution of the Plugsys system was one frame every 1.024 s including perfusion pressure (mmHg) and the (servo-controlled) flow (ml min^−1^). Vascular resistance was calculated by Ohm’s law. The Plugsys system also served as oxygenation- and pH-monitoring. Oxygen partial pressure in the venous outflow was kept over 150 mbar, pH was held constant between 7.30 and 7.45.

Luminal perfusion with 12 ml/min Krebs-Henseleit solution was performed by an Ismatec Reglo analog peristaltic pump (Cole-Parmer GmbH, Wertheim Germany). To keep the solution at 36.0–36.5°C it was operated over a separate heat exchanger. The luminal outflow was recirculated using a 500 ml reservoir. The maximum intraluminal pressure was set on 4 cmH_2_O by a riser tube 4 cm long above the bath level. To minimize its resistance, the outflow was equipped with a wide bore connector with a 7 mm inner diameter. The temperature of the Krebs Solution was held constant at 36.0–36.5°C. A Supplementary video provides a run-through of the entire experimental setup (Supplement 1). During transport and preparation, we kept the rectum segment in MEM at 4°C, the interval until the specimen was placed into the organ bath was 88 min ± 5 min (mean ± SEM), maximum 105 min. The application of the cold and readily prepared rectum segment temporarily lowered the organ bath temperature. During a 1-h adaptation phase, the temperature returned to 36–36.5°C. Vascular resistance was recorded every 1.024 s by the BDAS System for 20 min both after intra-arterial injection and in the control experiments. For graphical presentation, the arithmetic mean of 59 of those recordings was calculated in order to depict a smooth curve with one data point every minute. The rectum segment presented vivid motor activity which is exemplified in a Supplementary video (Supplement 2). The possible time window of experiments was limited by slowly increasing oedema beginning at around 4 h after perfusion commenced. Plasma expanders for the treatment of oedema have, however, been unsuccessful in pilot trials.

### Generation of neurospheres

Neurospheres were derived from the small intestine of mice according to an established method implemented in our lab [13]. Briefly, rat intestine was prepared immediately after euthanasia and stored in MEM (Life Technologies) containing antibiotics on ice. The muscle layer was dissected from the submucosa and was treated for 2 h with collagenase II (Worthington Biochemical Corporation, Lakewood, USA). We gained the myenteric plexus after vortexing, filtering through a sterile 40 µm cell strainer filter (BD biosciences, Franklin Lakes, USA) and centrifugation at 120 g for 5 min. The plexus particles were incubated in accutase (Sigma-Aldrich) for 20 min and dissociated by trituration. Accutase-containing media were removed after centrifugation 120 g for 5 min and replaced with expansion medium: Neurobasal A supplemented with 2% B-27 without vitamin A (Life Technologies), EGF 10 ng/ml (Cedarlane, Burlington, USA), bFGF 20 ng/ml (AnaSpec, Fremont, USA), GDNF 10 ng/ml (GenScript Biotech, Rijswijk, the Netherlands), 1% albumin (Sigma-Aldrich), 0.25% 2-mercaptoethanol 50 mM, (Thermo Fisher Scientific, Carlsbad, USA), 0.12% glutamine 200 mM (Sigma-Aldrich) and 1% penicillin/streptomycin (Thermo Fisher Scientific). The cell suspension was diluted to 1 million cells per 25 cm^2^ flask. After 2 days, the first floating neurospheres were to be observed. The neurons and radiating glial cells adhered to and expanded at the bottom [14].

### Preparation of the graft

For delivery, we centrifuged the supernatant medium of one flask containing the floating neurospheres at 200 g for 5 min. The cell pellet was resuspended in 10 ml PBS and incubated for 30 min with 10 µl of 10 mM fluorescein diacetate (FDA). The neurospheres were washed twice in PBS and resuspended in 5 ml MEM (Gibco, Thermo Fischer Scientific). The now fluorescein-labelled neurospheres were transferred to a 50 mm Petri dish and visualized with an Olympus IX 70 inverted microscope (Olympus, Tokyo, Japan) equipped with a FITC filter: excitation 470/30 nm, and emission 535/43 nm. Fifty 50–100 µm neurospheres were manually aspirated and transferred to a 2 ml syringe which was continuously rotated manually to prevent adhesion of the neurospheres. The volume of the suspension was around 1.5 ml depending on the manual harvesting procedure.

### Vascular injection

**Figure 2.**
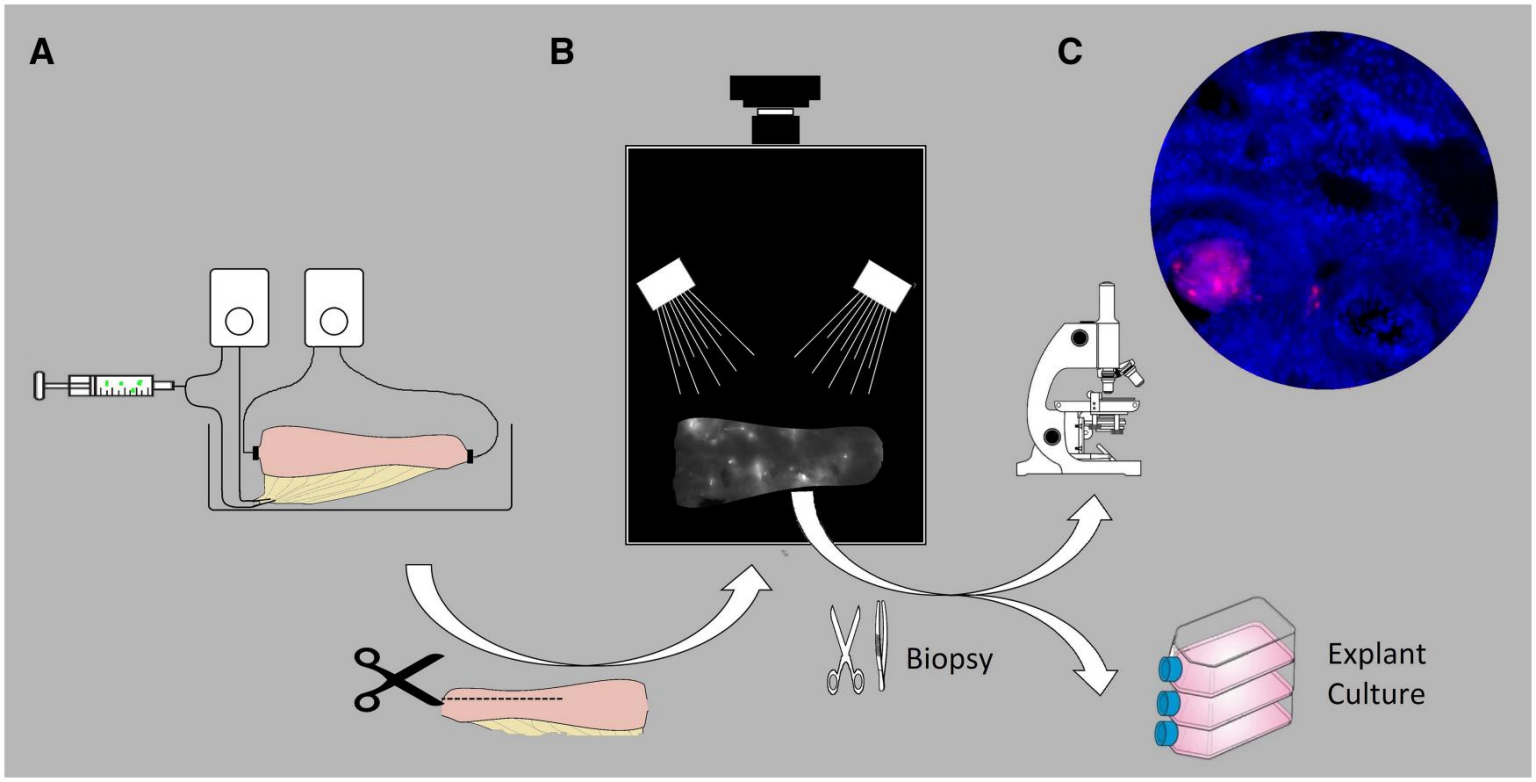
Schematic overview of the methodical concept of the experiments. (A) Fluorescein marked neurospheres were injected intra-arterially into a porcine rectum, which was perfused in an organ bath. (B) The longitudinally opened preparation was mounted to be analysed employing a fluorescence imager. (C) Biopsies were taken from the grafting sites and cryosections were examined using fluorescence microscopy. The biopsies are also kept as explant cultures to further study the engraftment in more detail.

After the adaptation phase of 1 h, the neurosphere suspension (1.5 ml) was injected by hand within 5–10 s into the inlet tube 15 cm proximal to the organ (Fig. 2A) into the arterial inlet of the perfusion system. During the injection, the perfusion pressure was held constant as potential overpressure could be balanced by a large, half gas-filled bubble trap 5-cm upstream of the injection site and by servo-controlling the roller-pump adjusted for constant-pressure perfusion. After 20 min of perfusion time post-injection, the organ was removed from the machine.

### Wholemount imaging and localization of single grafts

**Figure 3.**
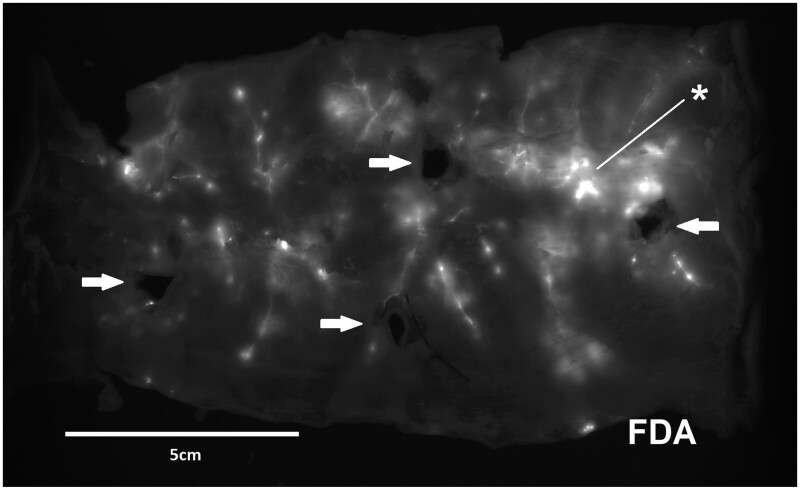
Result of the intravascular delivery depicted in a wholemount preparation of the specimen in a fluorescence imager (exposure for 20 s to 470/30 nm light and imaged through a 535/43 nm filter). Glowing areas indicate the grafting sites of single fluorescein (FDA)-labelled neurospheres (50 injected into the arterial inlet). Four black areas define the positions where biopsies have been taken (arrows). One central position with wide and intense fluorescence (asterisks) probably depicts the grafting of more than one neurosphere at one site.

The rectum segment was split longitudinally and mounted in a plane position with the serosa oriented towards the optical system of a fluorescence imager (ECL Chemocam, Intas Göttingen, Germany). The specimen was imaged by fluorescence microscopy for 20 s (excitation 470/30 nm; emission 535/43 nm). The grafting sites were identified as fluorescing spots (Fig. 2B). We marked fluorescing spots on the specimen by the tips of forceps around the spot. We used the vessel pattern identified in the fluorescence image for orientation on the preparation. Alternatively, we used x–y coordinates to localize the specific grafting sites. A further fluorescence image with the placed forceps confirmed correct targeting. We took one biopsy of each of the four identified grafting sites per experiment: 5 mm squares with the fluorescing spot in the centre were cut with scissors as full-thickness biopsies (dark areas, Fig. 3). These were immediately cryopreserved using n-pentane for quick freezing.

### Staining and microscopy

**Figure 4.**
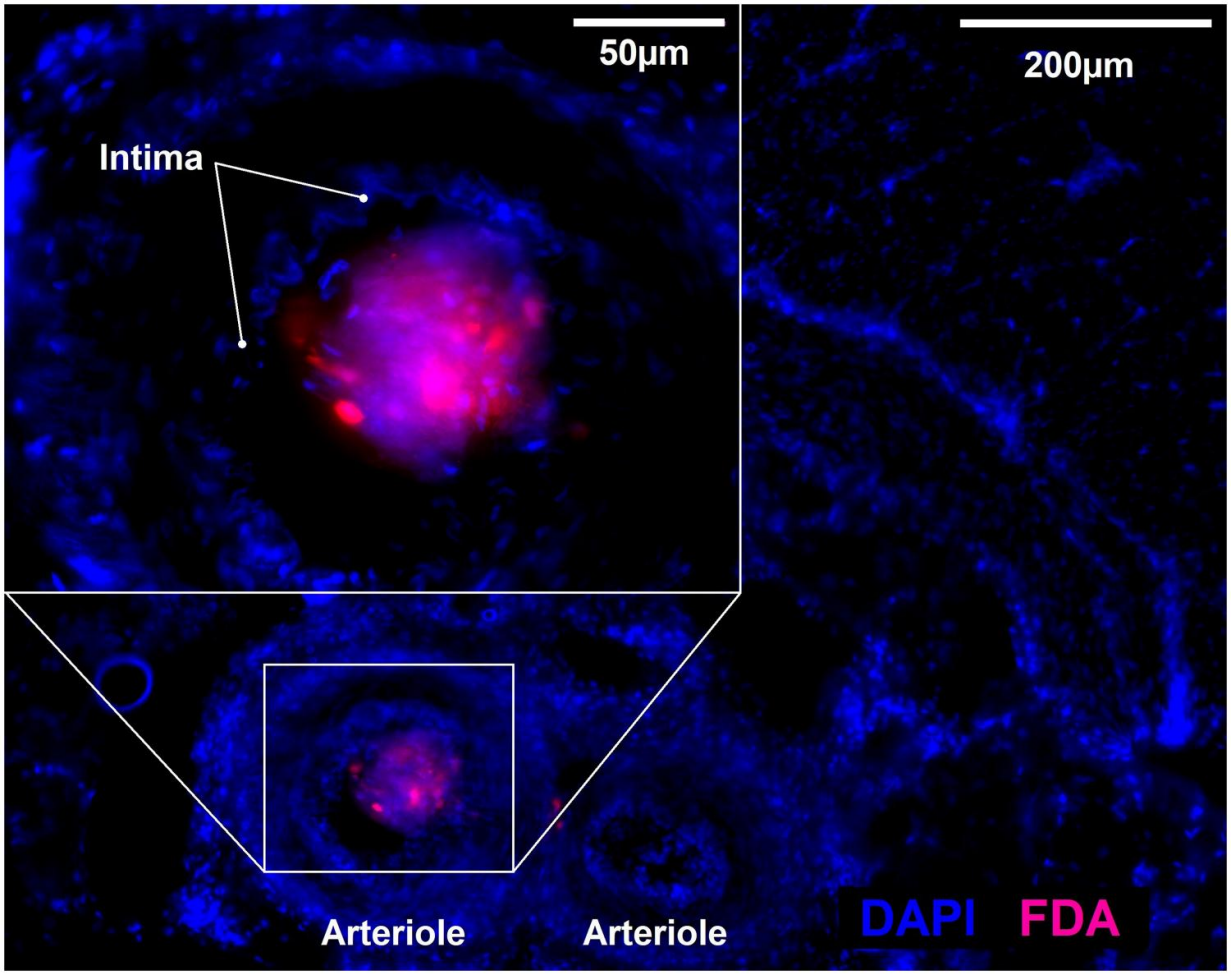
Cryosection of a biopsy from a grafting site stained with DAPI (blue) containing a fluorescein (FDA, red) marked neurosphere. The overview of the vascular bed demonstrates that fluorescence is originating from a single arteriole cross section. The inlay shows a detailed view of the arteriole lumen. Inside the clearly discernible intima, the fluorescence shows grafting of a neurosphere.

The cryopreserved specimens (5 mm × 5 mm × wall thickness) were sectioned throughout their entire volume with 100-µm thick sections employing a CM1900 cryostat (Leica, Nussloch, Germany). The specimen was oriented in the way that one 5 mm side and the wall diameter formed the section surface. Multiple 100 µm slides were mounted per object slide in a sequential arrangement. In the next step, the sections were fixed in ice-cold acetone for 10 min and then dried for 60 min. After Permeabilization with 0.5% Triton X-100 (Carl Roth, Karlsruhe, Germany) for 5 min, the sections were mounted with a Fluoromount-G mounting medium containing DAPI (Thermo Fisher Scientific). Microscopy was performed at an Axio Observer.Z1 (Zeiss, Jena, Germany) with filter sets 49 and 38 for DAPI (blue) and fluorescein, respectively (Fig. 2C). Every single section was scanned at 10× magnification for the detection of fluorescein. Detailed images at 40× magnification were taken from the fluorescent sites. For better recognition, fluorescein is depicted in red as false colour (Fig. 4).

### Statistical analysis

The vascular resistance was presented as mean ± standard deviation. The significance of intergroup differences was analysed by the Student’s *t*-test. The 20 arithmetic means over 59 data points (each representing one minute) in each individual course underwent serial unpaired *t*-tests over the whole 20-min observation period comparing the injection group and the control group. The statistical significance level was set to *P*-values <.05. The analyses were performed by the Statistical Analysis Software version 9.4 (SAS Institute Inc., Cary, North Carolina, USA).

## Results and discussion

Here, we describe novel methods for (i) monitoring the distribution of intra-arterially delivered neurospheres and (ii) localizing single grafts in a large porcine colon segment. In the past 20 years, intense research has been performed on cell replacement therapy for enteric neuropathies [13, 15, 16]. Within many studies, transplantation of either genuine human stem cells [5, 17] or neural crest-derived precursors of the enteric nervous system have been demonstrated in murine [4, 9] and rat [18] colons *in vivo* as well as human intestines *ex vivo* [6, 19]. Cooper *et al*. demonstrated that transplanted enteric neural crest cells proliferate and integrate structurally in aganglionic gut with projections closely associated with endogenous neural networks. They also show functional integration as proved by electrical point stimulation of transplants via donor nerve fibres [8]. However, there is still a need for an interventional method capable of reaching large colonic segments with human-size dimensions, as in a perfused porcine rectum segment. One approach to this is the transplantation of neurospheres [20, 21]. Taking the vascular route for clinical application would be feasible with state-of-the-art interventional catheter techniques. We understand our work as a technical step towards this application as we successfully grafted neurospheres after vascular injection in an isolated perfused porcine rectum *ex vivo*.

As we observed by fluorescence imaging, the injection of neurospheres into the arterial inlet spread the grafts over the whole colon segment in a disseminated manner: An example of the longitudinally split and planarly placed wholemount preparations of the 15-cm long porcine rectum segments is depicted in Fig. 3. Centrally, one position with wide and intense fluorescence probably marks the grafting of more than one neurosphere at one site.

In order to evaluate the transplantation of multiple grafts in the sub-millimetre dimension (e.g. neurospheres or organoids) into large organs, it is important to be able to localize single-delivered grafts and examine the grafting site by histology. We demonstrated, that it is possible to define the position of single grafts and take biopsies from these sites. The microscopic image displayed in Fig. 4 shows a 100-µm cryosection of a biopsy taken from a fluorescing spot of the porcine rectum. The detection of fluorescein-labelled cells (displayed in the false colour red) in the centre proves that the tissue biopsy has targeted one of the injected spheroid grafts. Stained with DAPI (blue), the intima of an arteriole is also clearly visible, indicating the intravascular position of the fluorescing neurosphere (inlay).

Monitoring the grafting success in terms of sprouting through the vessel wall into the tissue and functional integration of the intra-arterially delivered cells was, would take more time and was, thus, not part of this study. But certainly, the biopsies taken from the sites of delivery can be further examined for these parameters in an explant culture [19, 22]. The here described method is useful to avoid *in vivo* examinations (on pigs) to check the dispersion pattern of the grafting. Colonic preparations for this study were retrieved from precedent *in vivo* experiments of a neighbouring research group after the euthanasia of the pig. Hereby, the animals served more than one scientific purpose. Also, insufficient grafting is detected at a very early stage of a project, so that animals can be exempted from failed experiments.

**Figure 5.**
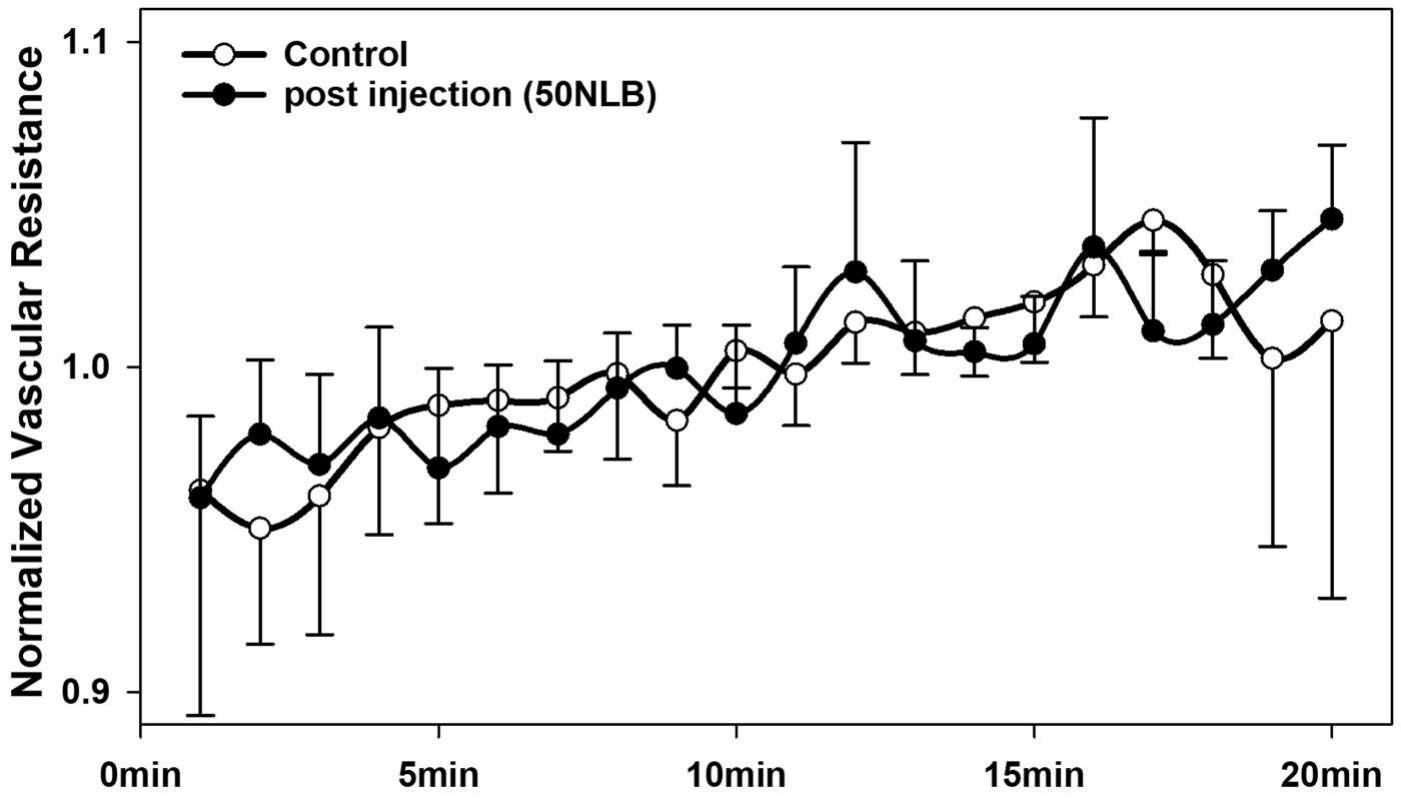
Course of vascular resistance after intra-arterial injection. 1-min data points show the geometric mean of the normalized vascular resistance in a 15-cm long isolated perfused porcine rectum segment after the intra-arterial injection of fifty neurospheres (50–100 µm) at time Zero (*n* = 3) and in control experiments (*n* = 3). The normalized values (dimensionless) were calculated for each individual experiment by division with the arithmetic mean of the individual course (mmHg/ml·min). No significant difference between any of the minute-values was detected by serial *t*-tests. Error bars indicate standard deviation.

A probable detriment of the proposed experimental therapy may be a circumscribed impairment of capillary perfusion in the downstream areas of the arterioles into which the neurospheres were delivered. During perfusion of the isolated rectum, the blocking of capillaries by the delivered neurospheres did not interfere with perfusion quality. The vascular resistance showed no significant difference between control experiments (*n* = 3) and experiments with the delivery of fifty neurospheres through the arterial inlet (*n* = 3) while perfusing in a constant-pressure mode over 20 min (Fig. 5). This aspect needs to be further examined in an *in vivo* study. The advantage of the presented method is that the consecutive risk for embolic damages can be evaluated before conducting *in vivo* experiments. At what level arterioles are embolized by the grafts and whether sufficient capillary perfusion is preserved from the surrounding has to be evaluated in pilot experiments performed *in vivo*.

## Conclusion

Injection of neurospheres into a perfused porcine colon segment over the arterial route disseminates grafted neurospheres over the whole colon segment. The usage of fluorescein diacetate and fluorescence imager allows for the localization of grafting sites for precise biopsy. The engraftment can be performed and monitored *ex vivo* and offers the opportunity to reduce animal experiments.

## Supplementary Material

bpae022_Supplementary_Data
